# Psychological, Emotional, and Neuropsychological Sequelae of Child Victims of Domestic Violence: A Review of the Literature

**DOI:** 10.1007/s40653-025-00746-6

**Published:** 2025-09-16

**Authors:** Natalia Bueso-Izquierdo, Mónica Guerrero-Molina, Carlos Barbosa-Torres, Mónica Ferrera-Silva, Juan Manuel Moreno-Manso

**Affiliations:** 1 Department of Developmental and Educational Psychology, Faculty of Psychology and Speech Therapy, University of Málaga, Málaga, Spain; 2 Department of Psychology & Anthropology, Universidad de Extremadura, Avd. Elvas, s/n, Badajoz, 06006 Spain

**Keywords:** Child victims, Domestic violence, Neuropsychology, Emotions, Psychopathology, Systematic review

## Abstract

Domestic violence is a global health problem with many consequences for mental health in children. Children’s exposure to domestic violence often begins at or before birth. This article highlights a summary of existing current evidence regarding psychopathological, emotional, and neuropsychological sequelae of domestic violence in child victims. Searches were conducted according to the PRISMA guidelines in PubMed, PsycNet, Google Scholar and Scopus to identify peer-reviewed articles reporting domestic violence and psychological, emotional and neuropsychological sequelae, yielding a final selection of 32 studies that met the inclusion criteria: (1) studies published in English; (2) carried out between 2000 and 2024; (3) reporting quantitative data, measuring their quality with the Newcastle-Ottawa scale. The results obtained were grouped into three tables with references, where the findings consistently indicated that children exposed to domestic violence suffer negative consequences that vary depending on the degree or magnitude of the violence to which they are exposed. Direct victims showed a greater degree of internalizing and externalizing problems, anxiety-depressive symptoms, emotional dysfunction, victimization, traumatic symptoms and problems in executive functions and cognitive performance compared to those children who had only witnessed the violence. We have also provided indications for future research and clinical practice because the data extracted from this review help to understand the consequences of experiencing or witnessing domestic violence, as well as the importance of early identification and intervention.

**Supplementary Information:**

The online version contains supplementary material available at 10.1007/s40653-025-00746-6.

## Introduction

Exposure to childhood violence is a major risk factor for multiple health problems worldwide (De Young et al., 2025; Prachason et al., 2024; van Baak et al., 2025). Experiencing trauma in childhood leads to the development of internalizing and externalizing symptoms, disruptive behaviors, and post-traumatic stress disorder (PTSD) (Cay et al., 2022; McLaughlin et al., 2012; McLaughlin et al., 2019). All of these consequences have been grouped into what are known as “adverse experiences in childhood and adolescence”. This model proposes different mechanisms for experiencing these events, which would have different consequences for the adaptation of these children (McLaughlin & Lambert, 2017).

Living in a home with DV is considered one of various possible adverse childhood experiences such as being a victim of psychological, physical, or sexual abuse. In the case of domestic violence, both parents can act as aggressors and victims (Künzle et al., 2022). The magnitude and severity of the problems associated with Domestic Violence (DV) extend beyond the couple, for adults are not the only ones affected. Children experience DV not only by directly witnessing physical abuse, but also by hearing screams, encountering damaged objects, or witnessing the emotional impact of violence.

As a result, children exposed to DV are at greater risk of emotional, social, and behavioral problems and its impact goes beyond their immediate reactions (Schubert, 2021), especially those under six years old, have direct sensory exposure to episodes of DV (Howell et al., 2010; Levendosky et al., 2003). In this regard, Künzle et al., (2022) found that children’s exposure to DV often began at or before birth, and approximately 40% of children had been exposed to violence for three years or more.

Meanwhile, Chemtob and Carlson (2004) found that 92% of children exposed to DV reported witnessing verbal abuse and 84% reported witnessing physical abuse of their mothers. In addition, 60% indicated that they themselves had experienced physical abuse and 4% had experienced sexual abuse. Similarly, the study by Ahmadabadi et al. (2018) of families with maternal Intimate Partner Violence (IPV) found that two out of five children were maltreated, compared to one out of five maltreated children in families without maternal IPV.

Another relevant longitudinal study by Johnson et al. (2002), which evaluated 167 children, found that more than a quarter (29%) of the children were categorized as victimized, one third had been physically victimized and more than three quarters had witnessed violence. However, caregivers tended to underestimate children’s exposure to violence. According to the caregiver self-report, they stated that more than three quarters (77.2%) of the children had been exposed to moderate-high levels of violence.

Scientific evidence has been provided that DV and child abuse are highly correlated (Ahmadabadi et al., 2018; Chemtob & Carlson, 2004; Turner et al., 2012), most of the research has focused on defining the characteristics of the perpetrators or the sequelae in direct victims, without taking into consideration that mere exposure to DV can have long-term consequences on children’s well-being and health (Künzle et al., 2022). There is extensive evidence that adversity and toxic stress are linked from childhood to later problems in learning, behavior, and physical and mental well-being in adults. We are talking about stressors and toxic variables that affect neurodevelopment such as: living through a war, experiencing poverty, discrimination or abuse in childhood, which can lead to the development of post-traumatic stress (Shonkoff et al., 2012). Exposure to traumatic events in childhood is often related to the family environment (Turner et al., 2012).

DV and child maltreatment occur within the context of close relationships and result in a variety of detrimental experiences, given that experiencing abuse by family members can give rise to significant trust and attachment issues. Consequently, an abusive family environment may be more traumatic for the child than other events outside the context of family relationships (Lapshina & Stewart, 2021). According to by Bender et al. (2022) highlights the impact of exposure to DV during childhood on the development of social and emotional competence and its potential to disrupt attachment relationships (Levendosky & Graham-Bermann, 2001), which provide a crucial environment for learning and practicing social and emotional skills.

The main consequences, apart from physical ones, are psychopathological, defined as cognitive, emotional, behavioral, or biological disorders in minors that impair their daily lives (Eldar et al., 2017). Therefore, psychopathological consequences include diagnosed disorders such as post-traumatic stress disorder (PTSD), depression, or attention-deficit/hyperactivity disorder (ADHD). On the other hand, emotional consequences encompass lasting emotional states that arise as a result of an event and hinder the daily lives of minors (Echeburúa, 2005), such as fear or worry. Finally, neuropsychological consequences refer to cognitive impairments resulting from brain damage (Sturm, 2007), such as deficits in concentration, memory, or executive functioning in a minor.

Throughout this work, psychopathological, emotional, and neuropsychological consequences have been distinguished because each affects different areas of a minor’s functioning and entails different intervention needs. While psychopathological consequences require clinical diagnosis and intervention, emotional consequences may necessitate interventions focused on emotional regulation, and neuropsychological consequences might demand more specific actions, such as cognitive rehabilitation. In conclusion, a differential analysis of the consequences in minors who are victims of domestic violence is considered beneficial and useful.

There has been an increased number of studies on the psychological and emotional consequences of children exposed to domestic violence (Clements et al., 2008; Evans et al., 2008; Kitzmann et al., 2003; Pearson et al., 2022; Ravi & Black, 2022; Wolfe et al., 2003). However, the absence of neuropsychological variables analyzed in previous research led us to implement a new approach. Therefore, this review will focus on exposing psychopathological and emotional variables, as well as the neuropsychological sequelae of child victims of DV.

## Method

A literature review was conducted considering the Preferred Reporting Items for Systematic Reviews and Meta-Analyses (PRISMA) guidelines (Page et al., 2021).

### Inclusion-Exclusion Criteria

The following criteria were used to determine whether the studies were eligible for inclusion: (1) studies published in English; (2) carried out between 2000 and 2024; (3) reporting quantitative data. We discarded reviews, PhDs, as well as works from degrees and masters. In addition, all the articles whose titles made no mention of the objective of this research, or the established key words were directly excluded.

Studies were excluded if the children reside in shelters because the term DV has been defined as assaults suffered within the family in the home where the child lives. In the specific case of children, DV can include direct violence against them and witnessing violence against other members of their family (e.g. their mothers or siblings), leading to sequelae and problems in the future for these children (Wood & Sommers, 2011).

The current study employs the term “psychopathology” to encompass the behavioral and/or cognitive manifestations of mental disorders. In turn, the term “emotional” refers to the effects of an event on the feelings or mental state of individuals or groups that results in a change in perception or behavior. Finally, the concept “neuropsychology” focuses on the brain-behavior relationships of individuals.

### Data Sources and Search Strategy

The search for articles to include in the review was carried out in the PubMed, PsycNet, Google Scholar and Scopus, for articles between January 2020 and March 2024 to identify studies that met the inclusion criteria. Combinations of keywords were used to run the search in the different databases as outlined below.

Firstly, different keywords and their combinations were used to create the following search equations: “children victims of domestic violence” AND (“psychopathology sequelae” OR “psychopathology consequence” OR “psychopathology”), “children victims of domestic violence” AND (“emotional sequelae” OR “emotional consequence” OR “emotional”) AND “children victims of domestic violence” AND (“neuropsychology sequelae” OR “neuropsychology consequence” OR “neuropsychology”). During the selection process, two independent reviewers (M.G.-M. and C.B.-T.) performed quality assessment and all duplicate articles were identified and eliminated. The titles and abstracts were then read to determine whether the articles met the inclusion criteria.

The references of eligible studies and relevant reviews were also searched using a snowballing technique. We thus obtained the following results from the various databases consulted: PubMed, 1880 studies; PsycNet, 48 studies; Google Scholar, 7866; and Scopus, 337. Having obtained a total of 10,127 results, we proceeded to eliminate those that were repeated or duplicated: due to finding the same article in different languages or the same work in different databases. Following this step, we had a total of 6079 articles, of which only 84 passed the established inclusion filters.

To decide whether these 84 articles were finally adequate for the review in question, their quality was measured using the Newcastle-Ottawa scale as additional bias assessment tools (Wells et al., 2000). This scale consists of a qualification system to compare studies based on such factors as design, analysis, or presentation. Having checked the quality of the articles, we then proceeded to a complete, exhaustive reading of the selected articles in order to take a final decision. Finally, articles that met the inclusion criteria after screening the title and abstract were retrieved and read in full to reach a final decision.

**Fig. 1:**
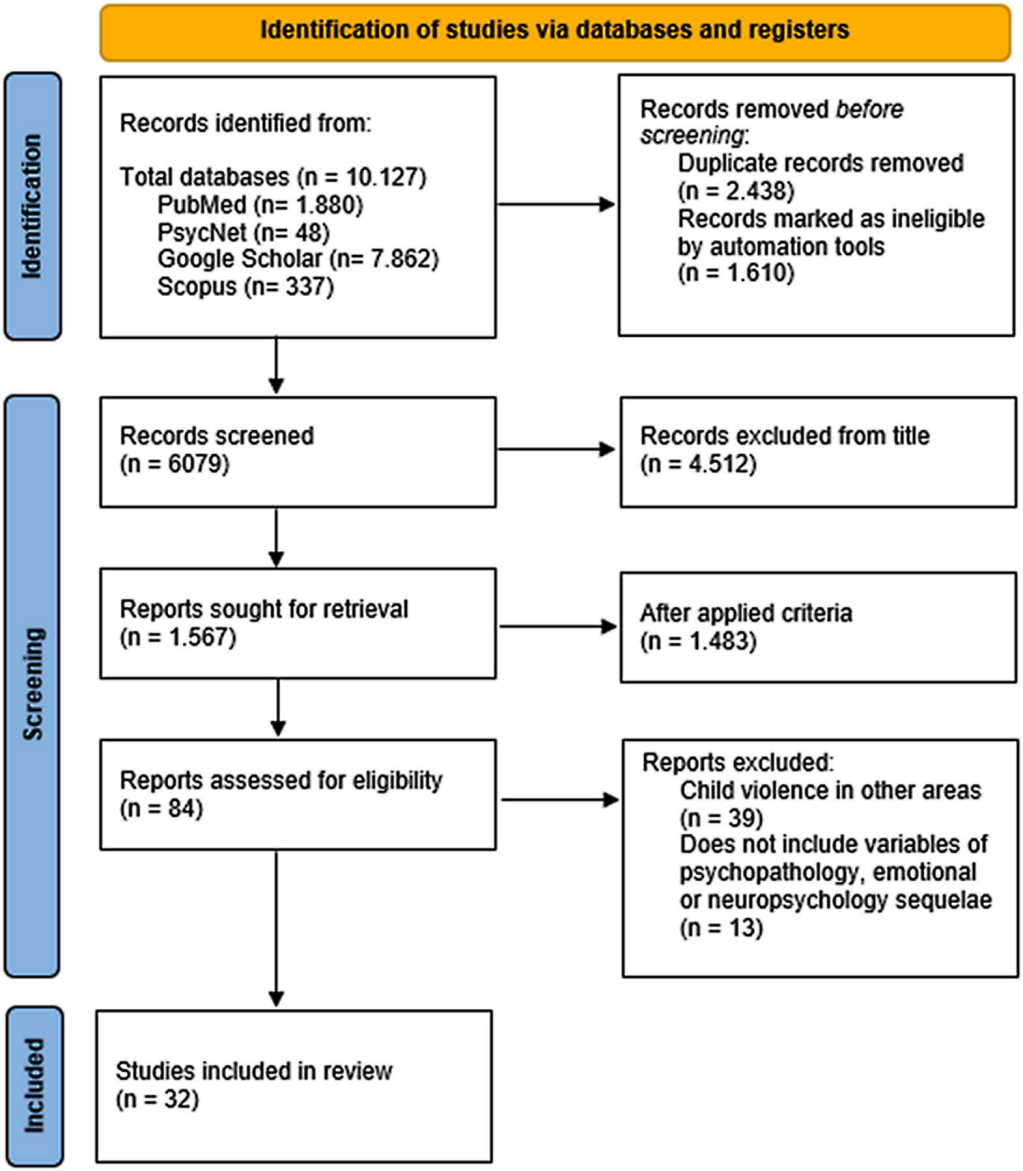
Identification and selection of articles for reviewing *Note*: PRISMA flow diagram of systematic search and study selection according to Page et al. (2021)

This complex process of identification, search and inclusion is clearly set out in the diagram of Fig. 1. Explicative flow diagram of the research method.

### Data Extraction and Analysis

A series of data were extracted from each of the articles: authors’ names, the number of participants including average age, instruments used to measure the results, and main results of the research. Only the results provided by standardized interviews and questionnaires were extracted from these studies.

## Results

The results of the review have been divided into three sections: the first section considers psychopathological variables, the second focuses on emotional sequelae and the third is related to neuropsychological variables. The main results are summarized in Tables 1, 2 and 3 respectively.

**Table 1:** Psychopathological sequelae in child victims of DV

Authors	Participants	Measures	Results
McCloskey and Walker (2000)	337 children aged 6–12 years.	- Coding of Stressor Responses. (Domestic Violence Episode. Violent Crime, Accidents. Death or Illness, Family History of Wife or Paternal Child Abuse) - Post-Traumatic Stress Symptom Clusters. - Child Assessment Schedule (to supplement the previous one) - Child Psychopathology - Children’s Self-Reported Feelings of Fear When Parents Argue	49% saw their father physically attack their mother and 12% reported having received high levels of physical abuse from their father. 15% of children suffered PTSD. An abusive family environment predicted PTSD. Children who met the PTSD classification criteria were more likely to show high levels of symptoms characteristic of phobias, with high rates of separation anxiety and oppositional defiant disorder.
Pelcovitz et al. (2000)	89 physically abused adolescents: *N* = 32 in families with IPV and *N* = 57 in homes with no reported IPV Control: *N* = 96.	- The Conflict Tactics Scale (CTS) - The Sexual Behavior Screen - The Family Disagreements Interview - The Kiddie Schedule for Affective Disorders and Schizophrenia (K-SADS-E) - The Family Adaptability and Cohesion Evaluation Scale (FACES III) -The Parental Bonding Instrument (PBI)	Adolescents with double exposure to violence in their homes are at greater risk of developing depression, oppositional defiant disorder (ODD), PTSD and SAD. Exposure to IPV was not a significant predictor of dysthymia, ADHD, conduct disorder or excessive anxiety disorder.
Levendosky et al. (2003)	103 children preschoolers	- Severity of Violence Against Women Scales (SVAWS) (parents) - Childhood Trauma Questionnaire (CTQ) (parents) - Social Support Quality of Life Scale - Life Stress Scale (parents) - Maternal psychological functioning [Beck Depression Inventory (BDI); Post-Traumatic Stress Scale for Family Violence] (parents) - Parenting Style Survey (parents) - Belsky’s (1989) parent-child interaction coding system.	DV has positive direct effects on both parenting efficacy and attachment, indicating that women who were more severely abused reported greater attachment on the part of school-aged children. More depressed and traumatized women reported lower parenting efficacy and more insecure attachment with their children.
Ybarra et al. (2007)	62 preschoolers aged 3–5 years (M age = 52.8 months); 31 children exposed to violence and 31 non-exposed children	- Screening Survey of Child Exposure to Community Violence (CECV) - Child Behavior Checklist (CBCL) - Revised Wechsler Preschool and Primary Scales of Intelligence (WPSSI-R) - Conflict Tactics Scales (CTS) - Life Stressor Checklist (LSC) - The Clinician-Administered PTSD Scale	DV-exposed children had a lower total IQ than non-exposed children. DV-exposed children showed higher levels of internalizing behaviors.
Briggs-Gowan et al. (2010)	213 children aged 24–48 months	- The PTSD section of the Preschool Age Psychiatric Assessment - Child Life Events Scale - PAPA	Exposure to violence was significantly associated with symptoms of depression, separation anxiety, post-traumatic stress disorder and ADHD (depression, SAD, PTSD, ADHD).
Bayarri et al. (2011a)	166 children aged 4–17 years; witnesses (*N* = 77), involved (*N* = 63), victims (*N* = 26).	- Schedule for the Assessment of Intimate Partner Violence Exposure in Children (SAIPVEC) - Diagnostic Interview for Children and Adolescents, (DICA) - Child and Adolescent Functioning Assessment Scale (CAFAS) - Child Behavior Checklist (CBCL) - Youth Self-Report (YSR)	The higher the level of children’s exposure to IPV, the higher the risk of psychopathology such as anxiety, depression, social problems, aggressive and rule-breaking behaviors and internalizing and externalizing symptoms.
Bayarri et al. (2011b)	144 children aged 4–17 years; witnesses (*N* = 72), involved (*N* = 52), victims (*N* = 20).	- Index of Spouse Abuse - Schedule for the Assessment of Domestic Violence Exposure in Children (SADVEC) - Diagnostic Interview for Children and Adolescents, (DICA) - Child and Adolescent Functioning Assessment Scale, (CAFAS) - Child Behavior Checklist (CBCL) - Youth Self Report (YSR)	Children who were direct victims of DV had more anxiety-depression, withdrawal-depression, somatic complaints, social problems, thinking problems, attention problems, disruptive behavior, aggressive behavior, internalizing problems, externalizing problems and general problems than children who only witnessed DV.
Miranda et al. (2013)	327 children aged 8–17 years.	- Schedule of Risk Factors (SRF) - Child Behavior Checklist (CBCL) - Child and Adolescent Functioning Assessment Scale (CAFAS) - Symptom Checklist 90 items - Revised	IPV correlated significantly with children’s externalizing problems and functional impairment.
Hagan et al. (2016)	211 children aged 3–6 years.	- Child Behavior Checklist for Ages 1.5-5 (Y-CBCL) - Trauma Symptom Checklist for Young Children (TSCYC) - Traumatic Events Screening Inventory-Parent Report Form, Revised (TESI-PR)	Post-traumatic stress symptoms and internalizing and externalizing symptoms were higher in severe exposure classes compared to moderate exposure and children who only witnessed violence.
Greene et al. (2018)	308 children aged 3–6 years.	- Revised Conflict Tactics Scale (CTS-2) - PTSD Checklist - Civilian Version (PCL) - Family Socialization Interview - Revised (FSI-R) - Preschool-Age Psychiatric Assessment (PAPA)	Maternal PTSD symptoms and parental behavior have a negative impact on symptoms of psychopathology in young children following exposure to IPV. Maternal PTSD was associated with internalizing and externalizing symptoms in children.
Weissman et al. (2019)	262 young people aged 8–16 years; 156 maltreated and 106 in the control group.	- Children’s Depression Inventory 2 (CDI-2) - Screen for Child Anxiety Related Emotional Disorders (SCARED) - Youth Self-Report (YSR) - Child Behavior Checklist (CBCL) - Childhood Trauma Questionnaire (CTQ) - UCLA PTSD Reaction Index (PTSD-RI)	The indirect effect of severity of maltreatment measured using ‘p’ (latent general psychopathology factor) during follow-up of emotional reactivity and rumination was statistically significant.
Adeyele and Makinde (2023)	664 children primary school	- Multidimensional Domestic Violence Scale - Child & Youth Mental Health General Screening Questionnaire	Findings showed a correlation between the children’s exposition to domestic violence and more rates of ADHD, ODD, AD, GAD and MD.

**Table 2:** Emotional sequelae in child victims of DV

Authors	Participants	Instruments	Results
Levendosky and Graham-Bermann (2001)	120 children aged 7–12 years.	- Conflict Tactics Scale (CTS). - Child Behavior Checklist (CBCL) - Children’s Depression Inventory (CDI) - Perceived Competence Scales for Children.	Child abuse rather than exposure to violence is the most significant predictor of children’s social and emotional adjustment.
Johnson et al. (2002)	167 children assessed at ages 6 and 8 years.	- Conflict Tactics Scales (CTS). - “Things I’ve Seen and Heard” survey. - Child Behavior Checklist (CBCL). - Trauma Symptom Checklist for Children (TSCC).	Both victimization and exposure to violence in children predict adverse internalizing and externalizing symptoms.
Chemtob and Carlson (2004)	25 children aged 7–17 years.	- Child Abuse Scale (CAS). - PTSD Scale for Children and Adolescents for DSM-IV (CAPS-CA).	Children are likely to experience emotional consequences directly associated with witnessing or experiencing DV.
Katz and Windecker-Nelson (2006)	130 children aged 4–5 years.	- Physical Violence subscale of the Conflict Tactics Scale (CTS). - Child Behavior Checklist (CBCL). - Meta-emotion interview.	Experience of DV was associated with less emotion coaching of anger and fear in children.
Overlien and Hydén (2009)	15 children aged 3–15 years.	- Interviews and group therapy sessions.	The most common reaction among children exposed to DV is to distance themselves emotionally.
Howell et al. (2010)	56 children aged 4–6 years.	- Revised Conflict Tactics Scales (CTS2). - Social Competence Scale (SCS). - Child Behavior Checklist (CBCL).	Exposure to IPV predicts lower emotional and social competence among children.
Turner et al. (2012)	2017 children aged 2–9 years.	- Juvenile Victimization Questionnaire (JVQ). - Children’s Perceptions of Inter-Parental Conflict (CPIC). - Trauma Symptom Checklist for Young Children (TSCYC).	Child exposure to DV is the factor most strongly associated with traumatic symptomatology.
Jouriles et al. (2019)	539 children aged 7–10 years.	- Revised Conflict Tactic Scales (CTS2). - Revised Children’s Manifest Anxiety Scale (RCMAS). - Children’s Disruptive Behavior Scale (CDBS).	Exposure to both sexual IPV and physical IPV are associated with externalizing symptoms in children.
Lapshina and Stewart (2021)	502 children aged 4–18 years with intellectual development disorder (IDD).	- Interview on potentially traumatic events (PTEs). - Externalizing Problems Scale.	Children with IDD exposed to DV are at greater risk of experiencing emotional and physical abuse and developing emotional and behavioral problems.
Schubert (2021)	149 children aged 2–17 years.	- Strengths & Difficulties Questionnaire (SDQ).	Children exposed to DV who do not receive treatment exhibit greater hyperactivity and more negative emotional symptoms and behavioral difficulties.
Spinazzola et al. (2021)	271 children aged 8–18 years.	- Developmental Trauma Disorder Semi-Structured Interview (DTD-SI). - Traumatic Experiences Screening Instrument (TESI).	Domestic violence victimization is associated with emotional and behavioral problems in childhood.
Künzle et al. (2022)	112 children aged 0–12 years.	- Semi-structured interview.	One third of children exposed to IPV experience symptoms of dysregulation of instinctual functions, developmental difficulties, learning difficulties and emotional and behavioral problems.

**Table 3:** Neuropsychological sequelae in child victims of domestic violence

Authors	Participants	Instruments	Results
Beers and De Bellis (2002)	14 children with maltreatment-related PTSD and 15 healthy comparison children aged 11–13 years.	- WISC-III. - Stroop Color and Word Test. - Wisconsin Card Sorting Test. - Trail Making B. - California Verbal Learning Test. - Rey-Osterrieth Complex Figure copy.	Children with maltreatment-related PTSD had worse neuropsychological performance.
Koenen et al. (2003)	1,116 monozygotic and dizygotic twins aged 5 years.	- Conflict Tactics Scale (CTS). - Wechsler Intelligence Scale (WPPSI): Vocabulary and Block.	Children who had experienced high levels of DV had IQs on average 8 points lower than the control group.
Jouriles et al. (2008)	69 children (34 girls and 35 boys) aged 4–5 years.	- Conflict Tactics Scale (CTS). - Alabama Parenting Questionnaire. - The Visual Reception scale and the Receptive Language scale from the Mullen Scales for Early Learning.	The results indicated that mothers’ positive parenting moderates the relation between children’s exposure to IPV and explicit memory functioning.
Enlow et al. (2012)	206 children aged 0–6 years.	- Interpersonal trauma (IPT) exposure. - Wechsler Adult Intelligence Scale.	Violence experienced during the first two years of life has significant and long-lasting effects on cognitive development.
Samuelson et al. (2012)	47 IPV-exposed children aged 7–16 years.	- Conflict Tactics Scale 2 (CTS2). - The Parent Perception Inventory (PPI). - Emotion Regulation Checklist (ERC). - The Wisconsin Card Sorting Test. - The Tower of London. - The Stroop Color-Word Association Test. - The Digit Span subtest of the Wechsler Intelligence Scale for Children.	Parenting and parental emotional functioning play an important role in children’s neurocognitive functioning.
Gustafsson et al. (2015)	154 families.	- Conflict Tactics Scale (CTS). - Stroop-like task. - Backward digit span task. - Flexible item selection task.	Early exposure to IPV is negatively associated with executive functioning in 5-year-olds.
Danese et al. (2017)	2,232 children monitored during childhood, adolescence and adulthood.	- Wechsler Adult Intelligence (WAIS-IV). - Wechsler Intelligence Scale for Children-Revised (WISC-R). - Wechsler Preschool and Primary Scale of Intelligence-Revised (WPPSI-R). - Cambridge Neuropsychological Test Automated Battery [CANTAB]) and processing speed (CANTAB). - Trail Making Test, part B).	Children exposed to violence in childhood had cognitive deficits in areas such as general intelligence, executive function, processing speed, memory, perceptual reasoning and verbal comprehension.

### Psychopathological Sequelae

Several studies have analyzed psychopathological sequelae in child victims of DV. The first study reviewed was by Ybarra et al. (2007), who analyzed the behavior of 62 preschoolers aged 3 to 5 years, of whom 31 children were DV-exposed and 31 children were non-exposed. The results showed a significantly lower level of cognitive functioning in the case of exposed children compared to their non-exposed peers. The exposed children had lower verbal and full-scale IQ scores on the WPPSI-R questionnaire than non-exposed children. In addition, the exposed children showed higher levels of internalizing behaviors (e.g., lowered self-esteem, anxiety, and depression). However, no differences were observed between the groups in terms of externalizing behaviors (e.g., aggression). Furthermore, children of mothers exposed to DV with greater psychological difficulties showed higher levels of internalizing symptoms than children of mothers with less psychological difficulties. Again, there were no differences with respect to externalizing symptoms. Finally, it was found that children exposed to higher levels of DV exhibited slightly more externalizing behaviors than children with low exposure.

A subsequent study by Briggs-Gowan et al. (2010) with 213 children aged 24 to 48 months showed that psychological disorders were more likely among children exposed to violence than non-exposed children (81.8% versus 40.0% respectively), with symptoms of depression, separation anxiety disorder (SAD), post-traumatic stress disorder (PTSD), attention deficit hyperactivity disorder (ADHD) and behavioral problems being significantly more common. According to these authors, exposure to violence was also significantly associated with anxious-depressive symptoms among parents. They also concluded that parental affective symptoms may partially or fully mediate the relationship between DV and children’s depressive and behavioral symptoms.

Three years later, Miranda et al. (2013) conducted a study assessing the mediator role of mothers’ mental health on child psychopathology in homes with IPV. One of the main findings of this study was that maternal childhood abuse correlated significantly with children’s externalizing problems, while IPV correlated significantly with children’s externalizing problems and with their functional impairment. Mothers’ mental health problems, expressed using a global severity index with variables such as depression, anxiety, and hostility, correlated significantly with children’s externalizing behaviors (Aggressive Behavior and Rule-Breaking Behavior Syndrome subscales) and internalizing behaviors (Anxious/Depressed, Withdrawn/Depressed and Somatic Complaint Syndrome subscales).

Greene et al. (2018) conducted a study with 308 children aged 3 to 6 years, finding that maternal PTSD symptoms and the behavior of the father (men) have a negative impact on young children’s psychopathological symptoms following exposure to IPV. In particular, psychological violence by the partner was associated with the externalizing symptoms (including oppositional defiant disorder, conduct disorder and attention deficit hyperactivity disorder symptoms) but not with the internalizing symptoms (including specific and social phobia, separation anxiety, generalized anxiety and depression/dysthymia) of the children. However, the more PTSD experienced by the mother, the more internalizing and externalizing symptoms were identified in the child victims. The results indicated that neither the physical nor the psychological IPV experienced by mothers was directly associated with the children’s symptoms. However, both types of victimization were directly associated with maternal PTSD symptoms. Finally, the results suggested that maternal PTSD symptoms mediated the relationship between mothers’ psychological and physical experiences of IPV, mothers’ restrictive/externalizing symptoms and children’s internalizing and externalizing symptoms.

In relation to the latter, a study by McCloskey et al. (2000) with 337 children aged 6 to 12 years showed that an abusive family environment and exposure to violence precipitated PTSD symptoms in children. In this study, 49% of the children witnessed their father physically attacking their mother and 12% reported receiving high levels of physical abuse from their father. One of the main findings was that exposure to DV was shown to be a significant risk factor for increased PTSD symptoms in children. In addition, the impact of direct victimization by the father gave rise to an even more pronounced significant result. Finally, it was observed that the children who met the PTSD classification criteria were more likely to show high levels of symptoms characteristic of phobias, with high rates of separation anxiety and oppositional disorder.

Another study by Pelcovitz et al. (2000) with 185 physically abused adolescents and their parents assessed the relationships between psychiatric disorders and exposure to DV and physical abuse. Two groups of physically abused adolescents were selected, of whom 57 lived in homes with IPV (double exposure) and 32 in homes without such violence (single exposure), and finally a third group of 96 non-abused adolescents living in non-violent homes (no exposure). One of the main findings when comparing the groups was that relative to the other two groups adolescents in homes with IPV had significantly higher rates of diagnosed disorders such as depression, oppositional defiant disorder, PTSD and SAD. Nonetheless, exposure to IPV was not a significant predictor of dysthymia, ADHD, conduct disorder or anxiety disorder for this group.

Bayarri et al. (2011a) conducted a study with 166 children aged 4 to 17 years divided into three groups: the first group consisted of children who had witnessed IPV (witness) (*N* = 77); the second group consisted of children involved in IPV (*N* = 63); and the third group consisted of children who were direct victims of IPV (*N* = 26). The results revealed that children are affected by IPV regardless of the type of exposure. However, children who were direct victims of IPV exhibited more mood disorders than children who were only witnesses and/or involved. Direct victims reported more anxious-depressive symptoms, thinking problems, aggressive behavior and internalizing and externalizing problems than those who only witnessed IPV. The study concluded that the higher the level of children’s exposure to IPV, the higher the risk of psychopathological symptoms, including anxiety, depression, social problems, aggressive behavior and rule-breaking.

The same author (Bayarri et al., 2011b) carried out another study with 144 children and young people aged 4 to 17 years, with several relevant results. When comparing psychopathological symptomatology according to the level of exposure to DV, it was found that preschoolers who were direct victims of DV had a higher incidence of sleep disorders (insomnia) than children who were only witnesses. Children who witnessed DV exhibited a greater presence of socially inhibited behaviors in their interactions (introversion). Finally, children who were victims of physical violence had a higher incidence of symptoms such as anxiety-depression, withdrawal-depression, somatic complaints, social problems, thinking problems, attention problems, disruptive behavior (rule-breaking), aggressive behavior and other internalizing and externalizing problems than children who only witnessed violence. Also, children who were involved in DV showed a higher presence of aggressive behaviors and externalizing problems than those who only witnessed violence. Both studies concluded that the higher the level of exposure, the greater the risk of psychopathology.

Hagan et al. (2016) conducted a study with 211 ethnically diverse children aged 3 to 6 years who had experienced or witnessed DV. The study showed that a considerable number of children had clinically significant psychological symptoms such as PTSD (54%), externalizing problems (25%) and internalizing problems (34%). Boys had a higher incidence of externalizing and internalizing symptoms than girls, although there was no gender difference in the clinical levels of PTSD symptoms. An Exploratory Latent Class Analysis was used to analyze the relationship between the variables through conditional response probabilities (CRP). Three models resulted from this analysis: the first model was characterized by the presence of DV and a low probability of experiencing other traumatic events; the second model was characterized by a higher probability of experiencing traumatic events, especially victimization; and the third model was characterized by a low probability of witnessing actual DV but a moderate probability of other events. With regard to the children’s symptomatology, the specific comparisons indicated that post-traumatic stress symptoms were higher in severely exposed children compared to those moderately exposed and witnesses. Also, internalizing and externalizing symptoms were higher in severe exposure types compared to cases of moderate exposure and/or children who only witnessed violence.

Three years later, Weissman et al. (2019) conducted a study which assessed the role of emotion regulation as a transdiagnostic mechanism linking child maltreatment with the emergence of psychopathology in a sample of 262 children and young people aged 8 to 16 years. A general psychopathology factor ‘p’ was estimated and compiled over two years, representing co-ocurrence of psychopathology symptoms across multiple internalizing domains (depression, anxiety and post-traumatic stress) and externalizing domains (attention problems, aggressive and rule-breaking behaviors). Its main conclusion was that young people with greater attention bias toward threat showed a greater increase in psychopathological symptoms over time. In addition, the indirect effect of the severity of the maltreatment on ‘p’ during parallel follow-up of emotional reactivity and rumination was statistically significant.

In the latest study reviewed, Adeyele and Makinde (2023) performed an investigation involving 664 children from a primary school in Nigeria. One of the main findings in this study was the observed correlation between domestic violence and increased rates of attention deficit hyperactivity disorder (ADHD), conduct disorder (CD), oppositional defiant disorder (ODD), general anxiety disorder (GAD), separation anxiety disorder (AD) and mood disorder (MD). In summary, the study demonstrates that children exposed to domestic violence are at a substantial risk of developing mental health disorders.

### Emotional Sequelae

Different studies have focused on analyzing the emotional sequelae of children exposed to DV in relation to different family characteristics. Levendosky and Graham-Bermann (2001) analyzed the factors mediating the effects of exposure to DV on children from an ecological perspective. The results accounted for 40% of the variance in children’s social and emotional adjustment, showing that child abuse rather than exposure to DV is the most significant predictor of children’s emotional adjustment. The results showed that psychosocial factors help to explain the effects of exposure to DV on children’s adjustment and functioning, as they are affected by DV due to the psychological impact of the abuser on the mother.

Chemtob and Carlson (2004) assessed 50 mothers and children in families with DV of a verbal, physical or sexual nature, with the child present in the home at the time of the abuse. The results showed high levels of abuse and symptomatology associated with the traumatic experience in both the children and their mothers.

Upon closer analysis of the results regarding the sequelae in children, the study found that the diagnosis of PTSD in mothers is not associated with the traumatic emotional sequelae experienced by the children. Chemtob and Carlson (2004) highlighted that evidence of these characteristics in children is not dependent upon the presence of psychopathology in mothers. The severe emotional dysfunction in these children is therefore likely to be due to witnessing or experiencing DV. Children with emotional sequelae from their exposure to DV were more likely to suffer angry outbursts both in the family and in the social context.

The study by Katz and Windecker-Nelson (2006) addressed the question of whether parents in domestically violent homes have difficulty talking to and helping their children manage their emotions (emotion coaching). They found that DV was not associated with a general deficit in emotion coaching in the home, but it was associated with less coaching of anger and fear. However, the emotion coaching moderated the relationship between DV and children’s behavior problems. Thus, when mothers coached their children there was no association between DV and children’s behavior problems. However, when mothers did not use emotion coaching DV was associated with higher levels of child behavior problems. Importantly, these effects were not attributable to the degree of DV present in the home, as there were no differences in DV between mothers who showed high and low levels of emotion coaching.

Furthermore, this study raises the possibility that coaching by parents may neglect the management of certain emotions in families with extreme levels of DV, as the children have difficulties modulating their experience of anger and fear. The study also suggests that families can avoid the impact of DV on children’s behavior through emotion coaching, for if parents in domestically violent homes have emotion coaching skills their children are less likely to be socially withdrawn (Katz & Windecker-Nelson, 2006).

On the other hand, Turner et al. (2012) studied the association between variations in family relationships and child trauma symptoms resulting from victimization and/or exposure to DV in a sample of 2,017 children. These authors concluded that the dysfunctional, inconsistent, and hostile parenting practices associated with violent homes generates severe emotional sequelae for children exposed to DV. In addition, they found a significant association between children’s trauma symptoms and emotional maltreatment, although witnessing DV had the most significant association with the children’s symptomatology.

Previous studies evidence that forensic consultation data of adult victims of IPV may provide information on the potential suffering of children exposed to DV, especially those who due to the lack of verbalization linked to their stage of development have difficulty communicating their suffering. However, in order to understand the emotional sequelae of children exposed to domestic violence it is necessary to listen to their stories.

In relation to the above, Johnson et al. (2002) conducted a longitudinal study to assess the extent to which internalizing and externalizing behaviors of 8-year-olds were related to witnessing violence in the home and/or victimization at the hands of caregivers before the age of 8 years. The results confirmed that children are negatively affected by victimization and violence they witness in their homes. More specifically, victimization was found to be a significant predictor of child aggression and depression, and witnessed violence was found to be a significant predictor of aggression, depression, anger and anxiety.

In conclusion, the authors found that children who had been physically abused exhibited more severe adverse behavioral and emotional outcomes compared to those who had not experienced any victimization. Furthermore, children who reported witnessing severe violence also had high anxiety and depression scores relative to the general population and to those who reported witnessing a minimal or moderate amount of violence in their social environment (Johnson et al., 2002).

Overlien and Hydén (2009) studied children’s actions during ongoing violent episodes, finding that children exposed to DV always do something in response to the violent episode. Based on coping theory, they found that the most common reaction of the children was to distance themselves emotionally from the violence. They also concluded that children oppose the violence, given that their way of responding never included accepting the violence or engaging in violent behavior.

Despite the multiple negative consequences associated with exposure to IPV, a substantial proportion of children showed evidence of resilience. The study by Howell et al. (2010) focused on prosocial skills and emotion regulation to assess whether violence severity had the potential to significantly impact the resilience of 56 children exposed to IPV within the past two years. The results indicated that less severe violence exposure predicted better emotion regulation and prosocial skill scores, which in turn were negatively correlated with maladaptive child behaviors.

Importantly, neither the child’s gender nor their ethnicity had a significant effect on resilience, so children of both genders and different ethnicities may be as vulnerable or equally resilient to the development of various adjustment problems after exposure to IPV. Thus, differences in resilience outcomes may be better accounted for by factors beyond individual characteristics of the child (Howell et al., 2010).

The study by Jouriles et al. (2019) examined whether IPV is associated with adjustment problems in children. Four IPV comparison groups were established: (1) nonviolent, (2) physical only, (3) sexual only and (4) sexual and physical. Children in the groups exposed to physical and/or sexual violence against the mother exhibited greater levels of externalizing problems than children in the nonviolent group, although the levels of externalizing problems among children in the physical-only and sexual-only groups did not differ. In addition, children exposed to physical violence exhibited more internalizing symptoms than children in the nonviolent group. The results suggest that children whose mothers reported experiences of sexual IPV have externalizing problems at levels comparable to those of children whose mothers experienced physical IPV.

Lapshina and Stewart (2021) examined the association between exposure to potentially traumatic events (PTE) and externalizing symptoms experienced by 502 children with intellectual development disorder (IDD). Notably, one of the three most prevalent PTEs among participants was witnessing DV (22%). In addition, exposure to DV was significantly associated with physical and emotional victimization of children, as well as externalizing problems. The study highlights that children exposed to DV are at greater risk of experiencing emotional and physical abuse and also of developing emotional and behavioral problems.

In relation to the above, Schubert (2021) notes that childhood exposure to DV predicts myriad negative long-term sequelae. In her study, she evaluated the effectiveness of a child witness domestic violence (CWDV) program using an intervention group (*n* = 69 children) and a control group (*n* = 80 children) consisting of children whose mothers received adult-focused domestic violence services. The results showed that those who received treatment showed less hyperactivity, fewer negative emotional symptoms, and fewer behavioral difficulties. These conclusions offer promising evidence of the impact of a psychoeducational group intervention on the trauma of domestic violence and the improved well-being of exposed children.

Research by Spinazzola et al. (2021) shows that victimization in the context of family violence has complex sequelae in childhood that are consistent with traumatic symptoms. In addition, they found that cumulative exposure to traumatic adversity in childhood such as physical assault or abuse, emotional violence, or exposure to DV is associated with multidomain emotional and behavioral problems.

More recently, Künzle et al. (2022) found symptoms of dysregulation of instinctual functions (sleep, appetite or crying), developmental difficulties (language and/or psychomotor difficulties) and learning difficulties (lack of concentration and memory problems), as well as emotional problems (sadness, fear and worry) and behavioral problems (agitation, aggression, nervousness and inhibition) in almost one third of the children (31%) of IPV victims.

### Neuropsychological Sequelae

Research carried out involving neuropsychological assessments of children who were victims of some type of violence during childhood shows that these individuals exhibit different sequelae in their neuropsychological functioning (Hart & Rubia, 2012).

One of the first studies conducted was by Beers and Bellis (2002), which evaluated 14 children with maltreatment-related post-traumatic stress disorder (PTSD) and 15 healthy comparison children who were similar to the PTSD patients in age, race, socioeconomic status and IQ. A neuropsychological assessment battery was administered to analyze changes in concentration, learning and memory. The results indicated that children who had suffered maltreatment and developed PTSD performed worse in measures of attention and abstract reasoning.

One year later, Koenen et al. (2003) researched whether DV had an impact on children’s intelligence by studying twins. The sample consisted of 1,116 5-year-old monozygotic and dizygotic twins who were administered IQ tests, with the children who had experienced high levels of DV scoring IQs which were on average 8 points lower than children who had not experienced any violence.

Jouriles et al. (2008) analyzed whether IPV experienced by mothers influenced the neuropsychological functioning of their children. 69 children were assessed, 34 girls and 35 boys with an average age of 5 years. The results showed that mothers’ positive parenting moderated the relationship between children’s exposure to IPV and explicit memory functioning. The study also found a negative relation between exposure to IPV and preschoolers’ performance on explicit memory tasks.

Subsequently, Enlow et al. (2012) conducted a longitudinal study with a group of children from birth through to 64 months of age who had been exposed to or experienced physical or emotional violence, abuse, neglect or sexual abuse or who had witnessed maternal partner violence. These authors found that violence experienced during the first two years of life had significant and long-lasting effects on cognitive development, even after controlling for socio-demographic factors such as gender, race/ethnicity, socioeconomic status, maternal IQ, birth complications, birth weight and cognitive stimulation in the home.

Samuelson et al., (2012) assessed 47 IPV-exposed children aged 7 to 16 years to examine the relationships between maternal emotion regulation, parenting and children’s executive functioning. The results indicated that parenting and parental emotional functioning played an important role in children’s neurocognitive functioning, helping to explain the mechanisms by which children exposed to IPV experience deficits in executive functioning. In addition, when comparing by gender, girls exhibited better executive functioning compared to boys.

Gustafsson et al. (2015) conducted an executive functioning study involving 154 families with children that examined the association between witnessing IPV when children were 24, 30 and 36 months old and their executive functioning when they were 60 months old. The results indicated that early exposure to IPV was negatively associated with executive functioning upon school entry at the age of 5.

Finally, Danese et al. (2017) conducted a longitudinal study to analyze whether a history of childhood violence victimization had an impact on impaired brain function. Cognitive functioning was measured in 2,232 individuals during childhood, adolescence, and adulthood. The results showed that individuals exposed to violence in childhood had cognitive deficits in areas such as general intelligence, executive function, processing speed, memory, perceptual reasoning, and verbal comprehension. Furthermore, they noted that all these deficits persisted during adolescence and adulthood.

## Discussion and Conclusions

Most research on DV has focused on the effects it has on the direct victims, although its consequences go beyond the incident and influence all family members (Ahmadabadi et al., 2018). Studies focusing on children exposed to DV document devastating effects on the children. To sum up, the research confirms that exposure to violence in the home is prevalent and constitutes a risk factor for children’s mental health (Adeyele & Makinde, 2023; Jouriles et al., 2019; Levendosky & Graham-Bermann, 2001).

Our review highlights that children who witness DV may suffer behavioral problems in the clinical range and have greater post-traumatic stress symptoms compared to other children (Howell et al., 2010). In this sense, children’s exposure to DV constitutes a form of psychological traumatization with a severe impact on functioning both within and outside the family, as studies report very high rates of PTSD among children exposed to family violence (Spinazzola et al., 2021). Similarly, minors exposed to IPV are more likely to develop conduct disorders (CD), attention-deficit/hyperactivity disorder (ADHD), generalized anxiety disorder (GAD), or symptoms of depression (Adeyele & Makinde, 2023; Briggs-Gowan et al., 2010). Existing literature links minors’ exposure to IPV with the development of Oppositional Defiant Disorder (Adeyele & Makinde, 2023). However, the literature highlights differences in outcomes depending on the type of exposure, as direct victims exhibit greater anxiety-depressive symptoms and both internalizing and externalizing problems compared to those who merely witness the violence (Bayarri et al., 2011a). Thus, it seems that child symptomatology is more strongly affected by conflictive interactions and adverse events and conditions, meaning that the absence of toxic family contexts may be a crucial factor to prevent psychopathology in children (Turner et al., 2012).

Although the existence of emotional sequelae in children does not correlate with the presence of disorders in mothers, affected mothers are less likely to seek mental health services for their children, highlighting the importance of programs designed to ensure that abused mothers demand early interventions for their children (Chemtob & Carlson, 2004). Likewise, Howell et al. (2010) found that mothers with fewer mental health problems, who may be better able to maintain a stronger attachment with their children, may be better equipped to help children master emotion regulation and develop prosocial skills.

In relation to the above, the literature shows that positive family characteristics are associated with positive child adaptation, even after exposure to DV, as they were negatively correlated with children’s externalizing behavior problems (Howell et al., 2010). This highlights the important need for independent psychological screening of mothers and children in families experiencing DV to determine the need for preventive mental health services. Given the descriptions of the extent and severity of various forms of maltreatment against mothers and their children, the conditions for the development of severe psychological disorders are present in these children (Chemtob & Carlson, 2004).

Minors exposed to IPV exhibit various neuropsychological consequences, such as poorer performance in attention, explicit memory tasks, and abstract reasoning compared to those who are not victims of IPV (Beers & De Bellis, 2002; Jouriles et al., 2008; Künzle et al., 2022). Similarly, a history of childhood victimization due to violence significantly impairs brain functions, resulting in lasting cognitive deficits in general intelligence, executive functions, processing speed, memory, reasoning, and verbal comprehension (Danese et al., 2017).

The studies reviewed show that children’s suffering generates post-traumatic symptomatology that can be difficult to detect. Therefore, parents need to be aware that DV can have a harmful impact on children’s health and well-being, and health and educational professionals should be encouraged to consider the possibility of DV when faced with such symptoms in children (Künzle et al., 2022; Schubert, 2021).

To sum up, the conclusion reached by the literature is that child exposure to DV has high potential for adverse outcomes as it increases the risk of emotional and behavioral problems. Overlien and Hydén (2009) propose modifying the usual concept of ‘exposure’ to ‘experience’, given that domestic violence is not something that children passively witness from a distance. Furthermore, they see children as competent informants in the sense that, apart from their caregiver’s stories, they have their own stories that will help us better understand the issue of children experiencing DV.

### Limitations and Future Directions

Certain limitations of the review process should be mentioned. Future studies should expand the search for articles in other languages, as there is specific literature on this topic in non-English languages (Alcántara-López et al., 2013). Similarly, it might be interesting to add studies on children in foster care who were previously victims of DV in order to be able to assess more participants in other settings. Another notable limitation has been the difficulty in discerning how to categorize the articles and place them within one of the three category blocks. To address this issue, we focused on the main objective of each article.

In the methodology, it would be beneficial to include, in addition to “children victims of domestic violence,” the term “intimate partner violence.” This inclusion would allow for a more comprehensive analysis of the issue, encompassing another term regarding its impact on children, thereby enriching the understanding of the phenomenon.

As a narrative review, this study presents some inherent limitations. Unlike systematic reviews or meta-analyses, narrative reviews lack definitive guideline statements, which may reduce replicability and introduce subjectivity (Sukhera, 2022). Also, it is important to acknowledge the impact of publication bias on the findings of this review. In this regard, studies with negative results or those that do not follow the expected trend are less likely to be published.

Another limitation of this review is the exclusion of doctoral theses. This aspect might restrict the breadth of available evidence, as theses often contain original research and innovative approaches not found in published articles. This could influence the comprehensiveness and depth of the findings.

Additionally, another limitation of this work could be related to the databases used, as incorporating other databases such as Web of Science might yield different results.

Similarly, it should be noted that not all the articles selected for the review possess the same methodological quality, which may affect the validity of the findings. Some of the reviewed studies involve small sample sizes (Chemtob & Carlson, 2004; Enlow et al., 2012; Ybarra et al., 2007), while others assess children based exclusively on maternal reports (Greene et al., 2018; Künzle et al., 2022). Likewise, there is methodological heterogeneity that complicates comparison between studies and may reduce the validity of general conclusions. For example, Pelcovitz et al. (2000) worked with a sample composed solely of Caucasian, middle-class families, whereas the study conducted by Weissman et al. (2019) included a sample mostly consisting of low-income families and children belonging to racial or ethnic minorities.

Likewise, there are inconsistencies across the different studies. Specifically, in the study by Levendosky et al. (2003), behavioral problems in children were observed only during mother–child interactions and not in other contexts. These findings are inconsistent with those of other studies, in which children exposed to domestic violence exhibited more behavioral problems across various domains (Briggs-Gowan et al., 2010; Miranda et al., 2013).

As a result of the review conducted, it would be appropriate to analyze how cultural context influences the experiences of minors, and even the reporting process of abuse, given that the studies included in the review come from diverse geographical regions. Thus, in future work, it would be valuable to examine the interaction between race/ethnicity, violence exposure, and children’s socioeconomic status. Similarly, future research would benefit from the use of multi-informant reports and direct observation of children’s behaviors.

Despite the findings highlighting evidence of psychopathological and neuropsychological consequences in minors exposed to domestic violence, we recognize the need to conduct longitudinal studies that address the long-term repercussions of such exposure, as proposed by Danese et al. (2017). Additionally, the use of mixed methods is recommended, complementing information gathered from families with in-depth interviews with minors, who serve as key informants of the abuse they have witnessed. Furthermore, it is crucial to examine the impact of violence on minors with disabilities, as only one of the reviewed studies considers this underrepresented population (Lapshina & Stewart, 2021).

The contributions of this review underscore the importance of intervention with these minors concerning the neuropsychological, psychopathological, and emotional consequences stemming from domestic violence. Exposure to maltreatment profoundly affects their personal, academic, and social functioning. To mitigate its long-term impact, therapeutic practices should not be exclusively centered on minors but also extend to victimized mothers, as their mental health significantly influences children’s emotional and behavioral regulation, reducing the likelihood of symptom manifestation (Chemtob & Carlson, 2004; Howell et al., 2010).

## Supplementary Information

Below is the link to the electronic supplementary material.

Supplementary Material 1

Supplementary Material 2

## Data Availability

Not applicable.
