# Supplementary material for: Psychological, Emotional, and Neuropsychological Sequelae of Child Victims of Domestic Violence: A Review of the Literature
Source: J Child Adolesc Trauma. 2025 Sep 16;19(2):485–500. doi: 10.1007/s40653-025-00746-6 (PMC13190954; doi:10.1007/s40653-025-00746-6)
Supplement: Supplementary file 2 — Supplementary Material 2 [file 40653_2025_746_MOESM2_ESM.docx]

# Newcastle-Ottawa scale for case-control studies

| Study | Selection, 4 points | | | | Comparability, 2 points | Outcome, 3 points | | | Total Score | Quality |
| --- | --- | --- | --- | --- | --- | --- | --- | --- | --- | --- |
|  | Selection of the cases | Selection of the controls | Definition of cases and controls | Comparability of cases and controls | Controls were selected based on a reference population | Assessment of outcome | Follow-up period | Outcome is assessed regardless of exposure |  |  |
| Table 1 |  |  |  |  |  |  |  |  |  |  |
| McCloskey & Walker (2000) | 1 | 1 | 1 | 1 | 1 | 1 | 1 | 0 | 7/9 | Good |
| Pelcovitz et al. (2000) | 1 | 1 | 1 | 1 | 2 | 1 | 1 | 0 | 8/9 | Good |
| Levendosky et al. (2003) | 1 | 1 | 1 | 1 | 1 | 1 | 1 | 0 | 7/9 | Good |
| Ybarra et al. (2007) | 1 | 1 | 1 | 1 | 1 | 1 | 1 | 0 | 7/9 | Good |
| Briggs-Gowan et al. (2010) | 1 | 1 | 1 | 1 | 1 | 1 | 1 | 0 | 7/9 | Good |
| Bayarri et al. (2011a) | 1 | 1 | 1 | 1 | 1 | 1 | 1 | 0 | 7/9 | Good |
| Bayarri et al. (2011b) | 1 | 1 | 1 | 1 | 1 | 1 | 1 | 1 | 8/9 | Good |
| Miranda et al. (2013) | 1 | 1 | 1 | 1 | 1 | 1 | 1 | 1 | 8/9 | Good |
| Hagan et al. (2016) | 1 | 1 | 1 | 1 | 1 | 1 | 1 | 1 | 8/9 | Good |
| Greene et al. (2018) | 1 | 1 | 1 | 1 | 1 | 1 | 1 | 1 | 8/9 | Good |
| Weissman et al. (2019) | 1 | 1 | 1 | 1 | 1 | 1 | 1 | 1 | 8/9 | Good |
| Adeyele & Makinde (2023) | 1 | 1 | 1 | 1 | 0 | 1 | 1 | 1 | 7/9 | Good |
| Table 2 |  |  |  |  |  |  |  |  |  |  |
| Levendosky & Graham-Bermann (2001) | 1 | 1 | 1 | 1 | 1 | 1 | 1 | 1 | 8/9 | Good |
| Chemtob & Carlson (2004) | 1 | 1 | 1 | 1 | 1 | 1 | 1 | 1 | 8/9 | Good |
| Katz & Windecker-Nelson (2006) | 1 | 1 | 1 | 1 | 1 | 1 | 1 | 0 | 7/9 | Good |
| Overlien & Hydén (2009) | 1 | 1 | 1 | 1 | 1 | 1 | 1 | 0 | 7/9 | Good |
| Howell et al. (2010) | 1 | 1 | 1 | 1 | 1 | 1 | 1 | 0 | 7/9 | Good |
| Turner et al. (2012) | 1 | 1 | 1 | 1 | 1 | 1 | 1 | 0 | 7/9 | Good |
| Jouriles et al. (2019) | 1 | 1 | 1 | 1 | 1 | 1 | 1 | 1 | 8/9 | Good |
| Lapshina & Stewart (2021) | 1 | 1 | 1 | 1 | 1 | 1 | 1 | 1 | 8/9 | Good |
| Schubert (2021) | 1 | 1 | 1 | 1 | 1 | 1 | 1 | 0 | 7/9 | Good |
| Spinazzola et al. (2021) | 1 | 1 | 1 | 1 | 1 | 1 | 1 | 0 | 7/9 | Good |
| Künzle et al. (2022) | 1 | 1 | 1 | 1 | 1 | 1 | 1 | 1 | 8/9 | Good |
| Table 3 |  |  |  |  |  |  |  |  |  |  |
| Beers & De Bellis (2002) | 1 | 1 | 1 | 1 | 1 | 1 | 1 | 0 | 7/9 | Good |
| Koenen et al. (2003) | 1 | 1 | 1 | 1 | 1 | 1 | 1 | 0 | 7/9 | Good |
| Jouriles et al. (2008) | 1 | 1 | 1 | 1 | 1 | 1 | 1 | 1 | 8/9 | Good |
| Enlow et al. (2012) | 1 | 1 | 1 | 1 | 1 | 1 | 1 | 0 | 7/9 | Good |
| Samuelson et al. (2012) | 1 | 1 | 1 | 1 | 1 | 1 | 1 | 0 | 7/9 | Good |
| Gustafsson et al. (2015) | 1 | 1 | 1 | 1 | 1 | 1 | 1 | 1 | 8/9 | Good |
| Danese et al. (2017) | 1 | 1 | 1 | 1 | 1 | 1 | 1 | 1 | 8/9 | Good |

*Note*. We rated the quality of the studies as low (0-3), moderate (4-6), or good (7-9).
